# A current-mode system to self-measure temperature on implantable optoelectronics

**DOI:** 10.1186/s12938-019-0736-0

**Published:** 2019-12-05

**Authors:** Fahimeh Dehkhoda, Ahmed Soltan, Nikhil Ponon, Anthony O’Neill, Andrew Jackson, Patrick Degenaar

**Affiliations:** 1 School of Engineering, Institute for Integrated Micro and Nano Systems, University of Edinburgh, Edinburgh, EH9 3JL UK; 2 NISC Group, Nile University, Al Sheikh Zayed, Giza, Egypt; 3 School of Engineering, Newcastle University, Newcastle, NE1 7RU UK; 4 Institute of Neuroscience, Faculty of Medical Sciences, Newcastle University, Newcastle, NE2 4HH UK

**Keywords:** CMOS sensor interface, Current conveyor, LED, Temperature sensor, Optogenetics

## Abstract

**Background:**

One of the major concerns in implantable optoelectronics is the heat generated by emitters such as light emitting diodes (LEDs). Such devices typically produce more heat than light, whereas medical regulations state that the surface temperature change of medical implants must stay below + 2 °C. The LED’s reverse current can be employed as a temperature-sensitive parameter to measure the temperature change at the implant’s surface, and thus, monitor temperature rises. The main challenge in this approach is to bias the LED with a robust voltage since the reverse current is strongly and nonlinearly sensitive to the bias voltage.

**Methods:**

To overcome this challenge, we have developed an area-efficient LED-based temperature sensor using the LED as its own sensor and a CMOS electronic circuit interface to ensure stable bias and current measurement. The circuit utilizes a second-generation current conveyor (CCII) configuration to achieve this and has been implemented in 0.35 μm CMOS technology.

**Results:**

The developed circuits have been experimentally characterized, and the temperature-sensing functionality has been tested by interfacing different mini-LEDs in saline models of tissue prior to in vivo operation. The experimental results show the functionality of the CMOS electronics and the efficiency of the CCII-based technique with an operational frequency up to 130 kHz in achieving a resolution of 0.2 °C for the surface temperature up to + 45 °C.

**Conclusions:**

We developed a robust CMOS current-mode sensor interface which has a reliable CCII to accurately convey the LED’s reverse current. It is low power and robust against power supply ripple and transistor mismatch which makes it reliable for sensor interface. The achieved results from the circuit characterization and in vivo experiments show the feasibility of the whole sensor interface in monitoring the tissue surface temperature in optogenetics.

## Background

Sensors play a vital role in many solutions in infrastructure monitoring, environmental monitoring and medical applications [1]. Implantable medical devices need to provide therapeutic intervention, but must also themselves ensure they do not cause harm through accidental electric discharge, overheating or degradation. As such, sensors are required to monitor the continuing (safe) status of the device. The challenge is when the sensor signal is both small and highly sensitive to other factors than the main physical parameter. Such factors can be compounded with the need to consume low power and frugal dimensions.

The target application for this work is optogenetics—a gene therapy technique to photosensitize neural tissue [2]. Typically, to operate effectively, optogenetically sensitized neurons need to be modulated with an irradiance of 0.7 mW/mm^2^. As tissue is not transparent, a very high-radiance source is therefore required in addition to genetic manipulation tools. Such a source should either be a high-radiance surface stimulator or an arrangement of penetrating optical probes which can deliver light close to the target neurons. An example of the former was developed for retinal prosthesis by Soltan et al. [3]. The latter case devices are typically called ‘optrodes’, and a specific example is provided by Ramezani et al. [4].

**Fig. 1:**
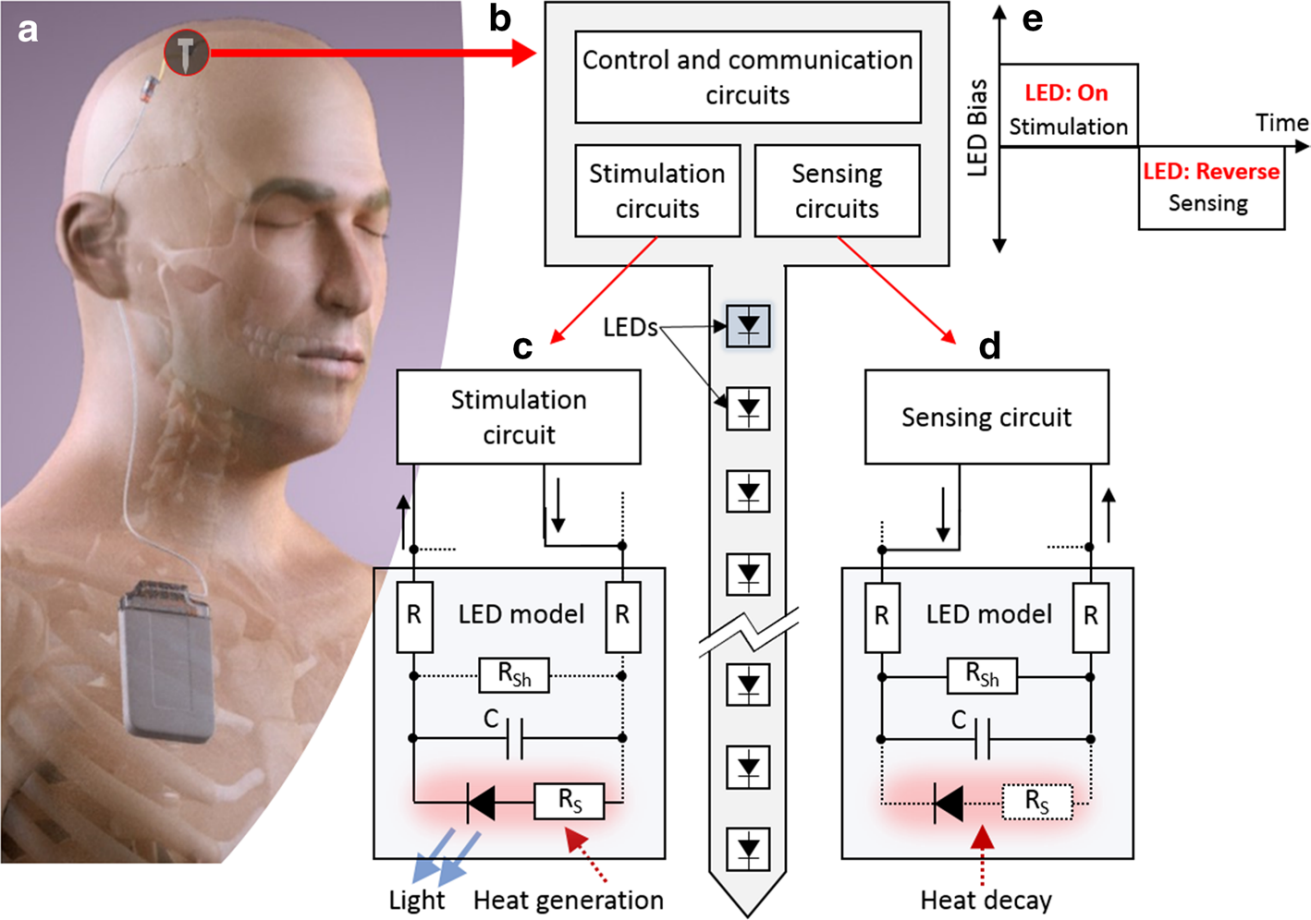
**a** Conceptual image of an implantable optoelectronic device; **b** concept optrode with LEDs on the shaft and stimulation and sensing circuits on the head, **c** LED in the optical stimulation phase generates heat; **d** reverse-biased LED connected to the sensing circuit to measure the reverse current change; and **e** timing diagram for the LED biphasic biasing

The concept of an optrode in tissue is shown in Fig. 1a. Electronics drive individual LEDs in specific depths which can then emit sufficient light to penetrate and stimulate target tissue. There are many different configurations of such devices, but typically they are formed from a silicon substrate, electronic control circuits and LEDs and some form of an encapsulant to allow for operation in tissue. Typically, LEDs emit more heat than light, as such, the proximity of the light emitting device to the tissue induces thermal effects [5, 6]. However, medical device regulations state that the surface of an implantable probe must remain less than 2 °C above normal tissue temperature [7, 8]. Therefore, one of the engineering design challenges in optogenetics is to develop a neural probe with additional functionality to monitor and control the generated heat in vivo [6].

We previously modelled the thermal emission of implantable light-emissive probes, the correlating results to infrared thermography [9]. We showed that thermal limitations could put significant constraints on the effective penetration depth of optical penetration. We, therefore, went further and explored the possibility of utilizing a temperature-sensitive device to monitor the LED surface temperature. Adding a resistance temperature detector (RTD) or thermistor would add to the required space on implantable devices [10–14]. As such, we explored the use of LED as their own temperature sensors by using the LED reverse current as a junction temperature-sensitive parameter (TSP). The correlation between the junction and surface temperature with the LED reverse current has been discussed in [15].

However, there are some challenges: (i) LEDs are typically constructed with multiple quantum wells, so their reverse current is highly nonlinear with voltage. Therefore, it is necessary to provide a robust, constant bias voltage across the LED during the sensing period. (ii) Measuring temperature with LEDs will provide their junction temperature, so a model is required to correlate the temperature of the junction with the surface. A detailed description of such modelling is given in [15].

Figure 1b shows a concept optrode with LEDs for optical stimulation and also temperature sensing. The LED in different phases and its timing diagram are shown in Fig. 1c–e which are explained more in detail in the results section.

Standard benchtop equipment allows for the measurement of thermally varying reverse currents while maintaining a stable voltage. Our objective here is to fit this capability on a small microelectronic circuit and integrate it onto a small microelectronic probe in tandem with other operations such as light emission and electronic recording, e.g. [4, 16]. Given that for current measurement at a stable voltage, current-mode circuits, utilized in instrumentation amplifiers, can be used. Such circuits are also more noise immune and able to work in low-voltage systems as such the signal representation is current instead of voltage [17, 18]. Options in the current domain include current conveyors [19], current-mode amplifiers [20, 21] and current mirrors. All can buffer currents, but current conveyors can be superior in terms of defining specific and stable voltages at the input contacts over the range of sensor operation.

The CCII (second-generation current conveyor) circuit has been used in many different applications. Examples include filters [22], wideband waveform generator and oscillators [23, 24] and other instrumentation systems [25, 26] which show particular relevance to sensor interfaces. The CCII advantage over corresponding op-amps can be higher voltage gain over a larger signal bandwidth. It can also provide better CMRR in instrumentation amplifiers, but non-ideal impedance at X and Z terminals can limit the CCII performance [27]. The main challenges in designing CCII can be the distortion introduced by a mismatch of the transistors in the CCII’s current mirror [28, 29] and the requirement for dual voltage supplies which is undesirable in portable systems [30, 31]. There are several reports describing CCII-based sensor interface circuits [32, 33]. Nevertheless, at the time of writing, there are only a handful of papers targeting low-power portable or bio-implantable devices for physiological monitoring [34, 35].

To overcome the bias voltage challenge, a CCII interface with low-power operation has been developed to measure the LED reverse current variation and convert it to junction temperature variation while providing a robust and constant bias voltage across the LED. A calibration method is employed to determine the surface temperature variation based on the measured junction temperature change [15]. In this work, we demonstrate a capacity to sense over a broad range of 12 °C and utilize the technique to specifically explore the surface temperature change on a LED optrode as a function of LED drive current.

## Results

The developed sensor interface circuit including H-bridge, CCII and TIA were experimentally tested using a Keithley 2612B source measure unit and an Agilent digital multimeter 34460A, while the AC and harmonic measurements were taken using an Agilent oscilloscope MSO-X4034A. The chip has been experimentally characterized, and its temperature-sensing functionality has been explored by interfacing different mini-LEDs in saline models of tissue prior to in vivo operation.

### CCII characterization

**Fig. 2:**
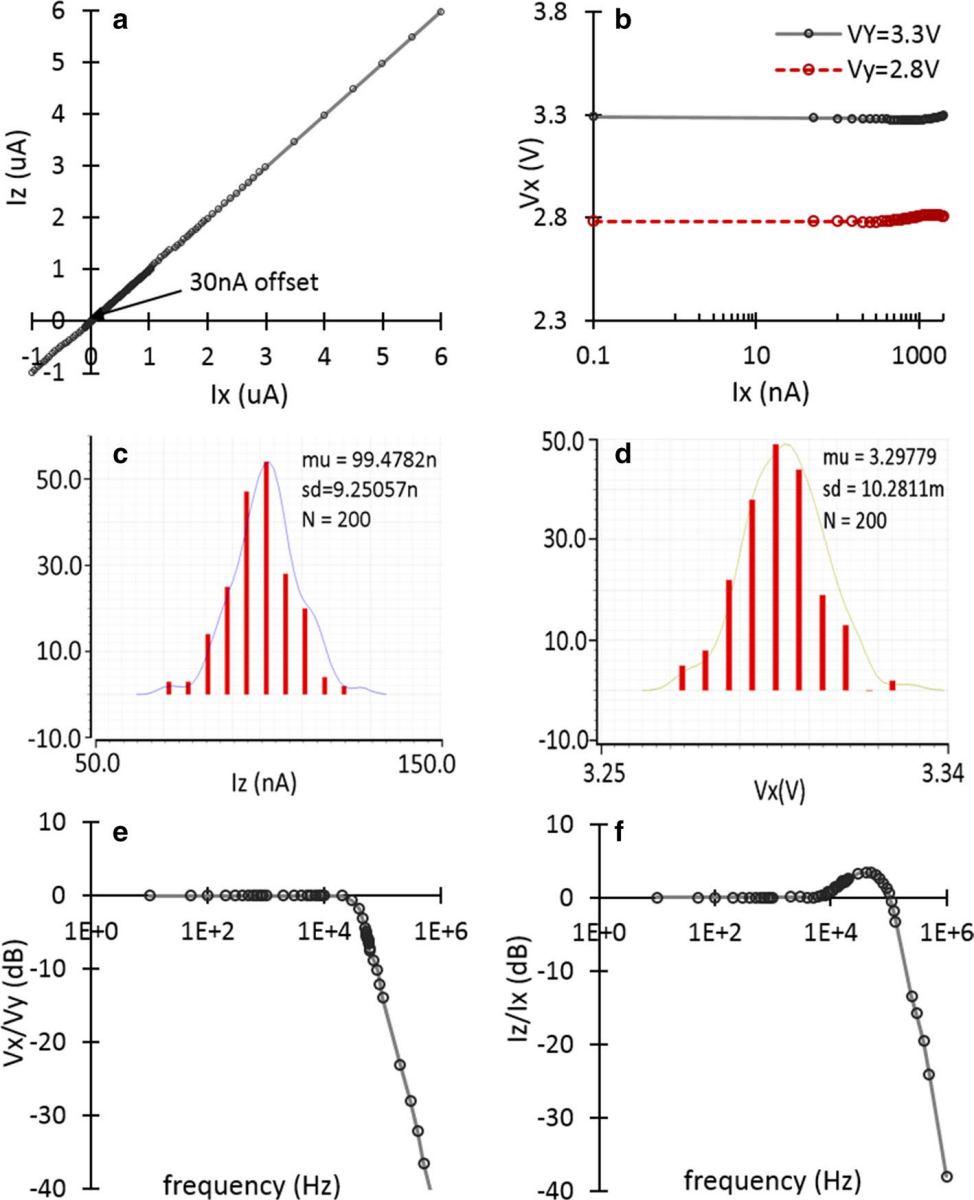
Experimental results; **a** linear current transfer characteristics of the CCII; **b** measured voltage at X terminal versus the input current variation in two different *V*_Y_ voltages, 2.8 V and 3.3 V; **c**, **d** Monte Carlo post-layout simulation results showing *I*_Z_ when *I*_X_ = 100 nA and *V*_X_ when *V*_Y_ = 3.3; and **e**–**f** measured voltage and current frequency responses of the CCII with cut-off frequencies at 45 kHz and 130 kHz, respectively

The designed CCII has been characterized experimentally. The measured current gain from X to Z is illustrated in Fig. 2a. The unity current gain was achieved in the range of − 1 μA to 6 μA, which covers the output current range of the employed LEDs. The output current has a 30 nA offset which has been achieved for every chip under the test (seven chips in total). This offset can easily be removed in the processing stage. Figure 2b shows the measured voltages in two different experiments where the voltage buffer was characterized in the presence of a current variation. In each experiment, a different bias voltage buffered from Y to X has been measured at terminal X while sweeping the received current at X terminal from 100 pA to 1 µA. The results show a stable unity gain voltage transfer function from Y to X in the presence of the current variation at X. This proves that the provided bias voltage across the LED is fixed and robust during sensing procedure. Hence, using this configuration, the LED reverse current variation is due to the thermal variation only and independent from voltage change. The measured Δ*V*_X_ for a fixed *V*_Y_ is 1%.

The transient response of the circuit has also been explored. The measured total harmonic distortion (THD) was 0.01% for low frequencies and 2% for mid-band and high frequencies. The power supply rejection ratio (PSRR) was also of particular importance as it can affect the circuit bias and performance. The measured PSRR is 57 dB when the power supply has 28% ripple. This shows a minimal effect of the power supply variation on the circuit. The effect of the transistor mismatch is explored using Monte Carlo (MC) analysis. Figure 2c, d shows the post-layout MC simulation results to estimate the mean values and variations in the conveyed current from X to Z and the buffered voltage from Y to X as a result of mismatch. The frequency response of the CCII has also been experimentally explored. Figure 2e, f shows the measured frequency response of the voltage and current transfer functions for the CCII circuit. The cut-off frequencies are measured at 45 kHz and 130 kHz, respectively. They are limited by the bias currents which are low to decrease the power consumption.

The mismatch effect has also been explored experimentally by measuring the circuit key features in different chips. Table 1 lists and compares the current and voltage gains achieved from MC simulations and the experiments carried out on seven different chips. The results show similar mean values for current and voltage gain of the circuit achieved by simulation and experiments. It should be noted that the current gain approximates to 1 over 4 decimal places. As such, it can be used to accurately account for temperature and even for systems which are highly sensitive to voltage variation.

**Table 1:** Mismatch for CCII current and voltage DC gains based on the Monte Carlo post-layout simulation and experimental results

Performance	MC simulation results		Experimental results	
	Mean	StDev (%)	Mean^a^	StDev (%)
Current gain	0.9947	9.24	1.0033	0.13
Voltage gain	0.9997	0.28	0.9826	0.15

^a^Based on the experiments carried out on seven different chips

### Temperature sensor operation and calibration

**Fig. 3:**
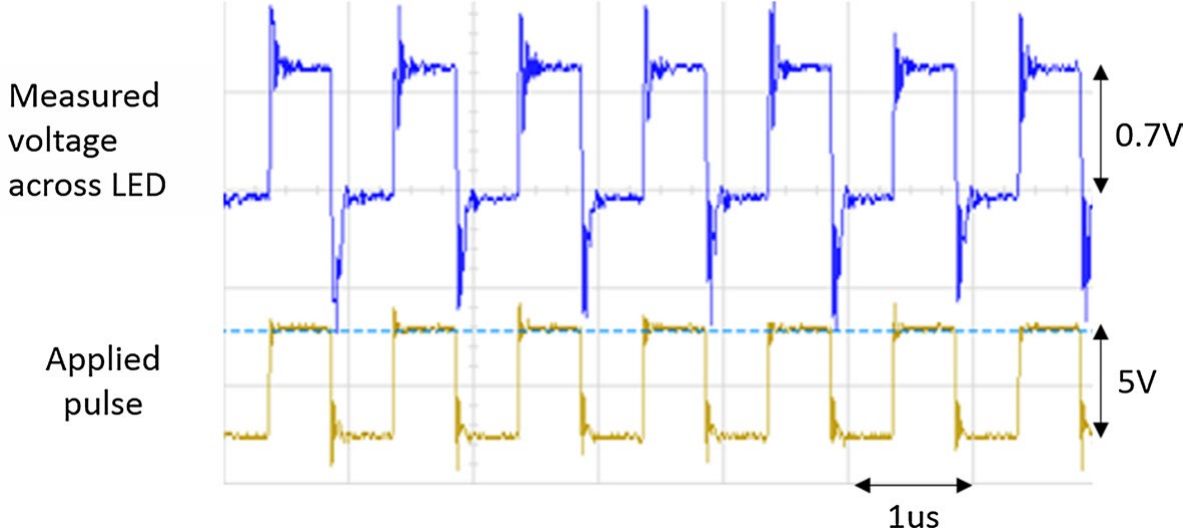
Transient response of the LED switching ON and OFF with a clock signal of 1 MHz. The blue pulse shows the measured voltage across the LED while the yellow pulse was applied as input signal. The spikes on the output signal are mainly because of the LED, and the shorter spikes on the input pulse are introduced by the function generator

In order to assess the heat generation on the LED surface and the sensor interface functionality, a two-phase operation (stimulation–temperature sensing) has been carried out in our experiments. The control signals switch the LED to forward bias using *S*_*F*_ switches and then to the sensing phase using *S*_*T*_ switches. In forward bias, the LED is ON for optical stimulation, and therefore, temperature increases by time (Fig. 1c). In the sensing phase (Fig. 1d), the LED is biased using a certain reverse bias voltage and its reverse current as TSP is measured using the sensor interface. The measured current variation is then converted to junction temperature variation and calibrated to achieve the change in surface temperature. To accurately perform this, the system clamps a fixed and robust reverse bias voltage across the LED to differentiate between temperature and voltage variation. The measured voltage variation is within ± 5% of a target bias voltage. Figure 3 depicts the capability of the employed LED in switching between ON and OFF states at 1 MHz to show that LED does not affect the time constant of the system. The spikes on the output pulse (measured across the LED) are mainly due to the parasitic capacitance of the LED and the small spikes on the applied pulse are introduced by the function generator. Thanks to the MHz bandwidth of LEDs, they can be used in fast switching application [3, 36].

**Fig. 4:**
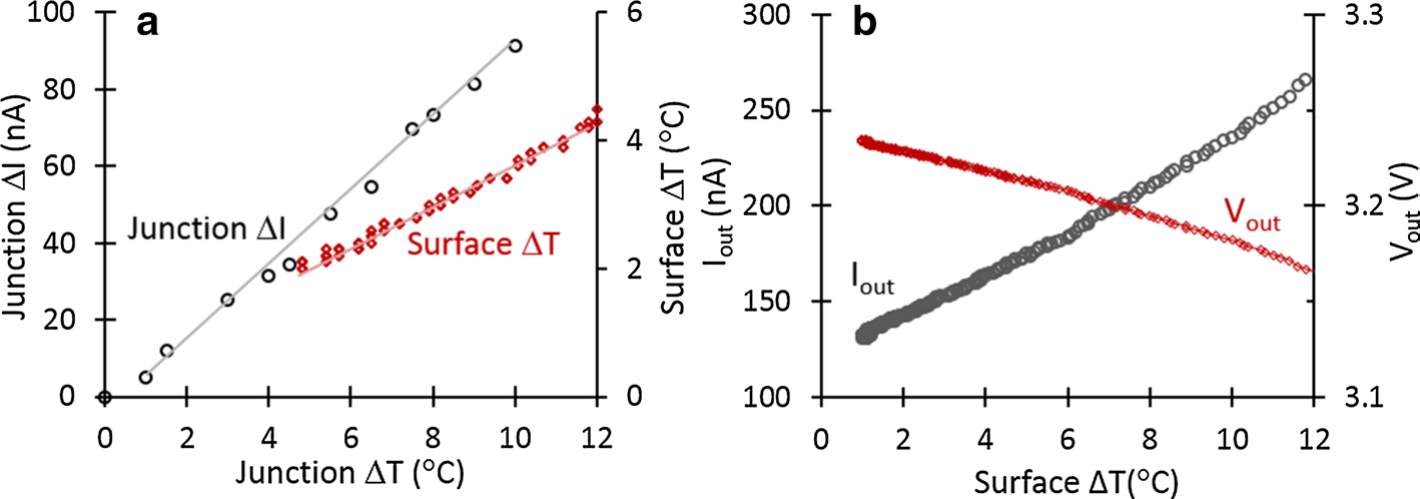
**a** Measurement results showing the linear relationship between junction current, surface temperature and junction temperature variation while the LED was driven using 2.5 mA pulse in water and the base temperature was 37 °C (**b**) measured output current and voltage of the sensing system which are linearly changing with surface temperature of LED driven using 7.5 mA pulse in water

Using LED as a temperature sensor will measure the junction temperature rather than the surface temperature. There will be a temperature gradient between the two which will need to be calibrated. The process for achieving this was explained in our previous work [15]. To summarize, Fig. 4a shows the measured LED current and surface temperature change versus its junction temperature variation after driving the LED using 2.5 mA pulse in water. The base temperature has been fixed at 37 °C, and thus, we are interested in variances from that rather than absolute temperature. These linear relations are used for calibration and achieving the relation between the LED reverse current and surface temperature.

A different experiment was performed by driving the LED in water with 7.5 mA pulse. Figure 4b depicts the sensor interface output current and voltage variation versus the LED surface temperature change achieved after applying the calibration method on the measured current data. The measured temperature sensitivity is 5–10 mV/ °C, which is dependent on the type of the employed LED.

**Fig. 5:**
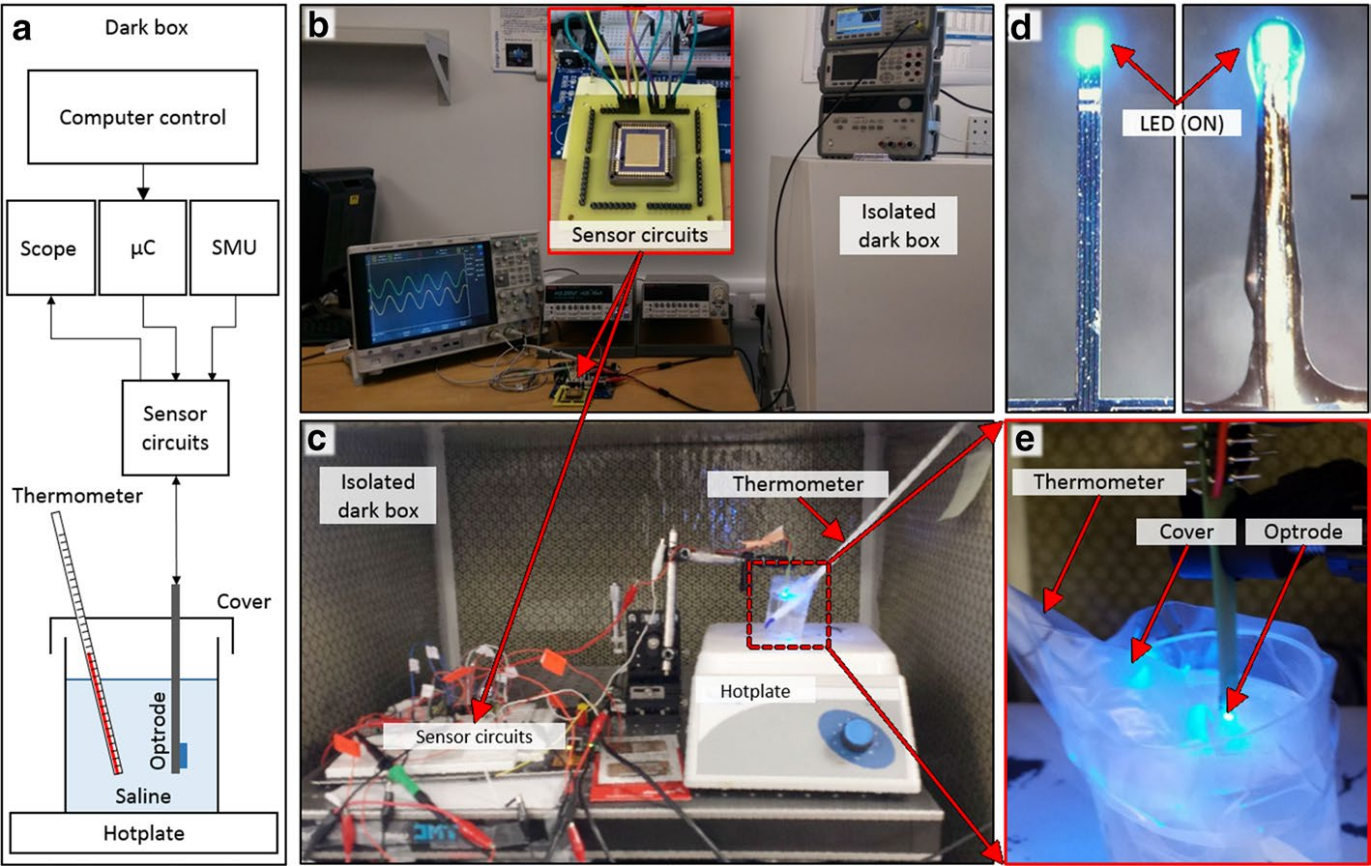
**a** Diagram of the set-up inside a dark box for measuring the temperate of an illuminating LED in saline to use for calibration. The hotplate is to maintain 37 °C temperature and a thermometer monitors the temperature during the experiment; **b** experimental set-up to electrically characterize the circuits; **c** image of the experimental set-up inside the dark box; **d** image of non-encapsulated and encapsulated mini-LEDs employed in the experiments; and **e** close view of the optrode with illuminating LED dipped in water

Figure 5a shows a block diagram of the water experiment in which the CMOS electronics control the LED dipped in water. The water container is placed on top of a hotplate to keep the temperature constant at 37 °C during the experiment, and a thermometer measures the temperature change after pulsing the LED. The image of the set-up for the electrical characterization and calibration which is illustrated in Fig. 5b,c. Figure 5d shows the non-encapsulated and encapsulated mini-LEDs used for the experiment.

**Fig. 6:**
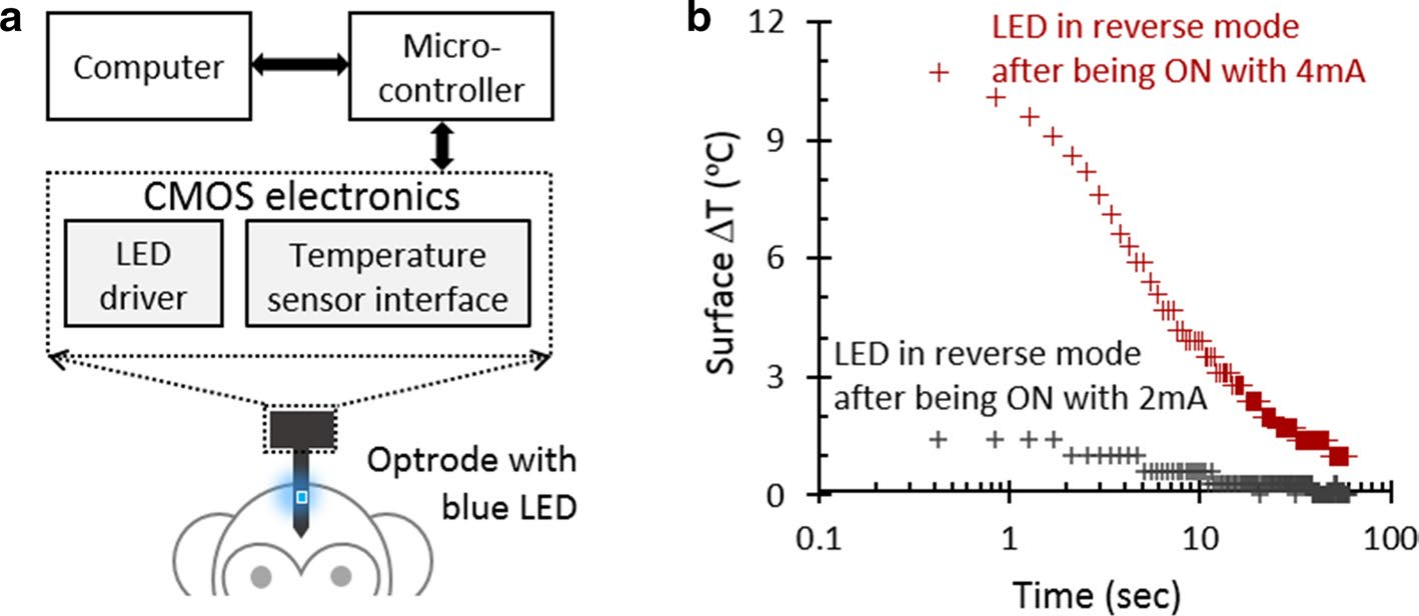
**a** Developed non-human primate experimental set-up with an inserted optrode into brain tissue. A blue LED has been bonded on the optrode shaft; **b** extracted surface temperature variation during the sensing phase after driving the LED with 2 mA and 4 mA currents for a minute to have optical stimulation. The LED reverse current is measured after switching the LED to reverse bias while cooling down. The temperature data are extracted by applying the calibration method to the measured current and junction temperature

Figure 6a illustrates a diagram of the developed set-up for non-human primate (NHP) experiment to explore the functionality of the temperature sensor in optogenetic. The optrode has been fabricated according to our previous work [15] and encapsulated with blue LED on the shaft inserted into non-human primate (Macaca Mulatta) brain to assess the temperature rise during optical stimulation. The LED on the optrode was inserted to a depth of 2–3 mm into prefrontal regions of cortex. The benchtop source measure unit (SMU) and microcontroller supply and control the CMOS electronics, respectively. The LED is driven using 2 mA and 4 mA currents for a minute to have long-term optical stimulation on the brain tissue and generate heat. The LED is then switched to the sensing phase where the CCII maintains a fixed bias voltage across the LED and transfers its reverse current for amplification and conversion to perform thermal sensing. In both experiments, the CCII provides a same stable reverse bias voltage across the LED to measure the reverse current change only due to the junction temperature rise.

Figure 6b illustrates the surface temperature variation extracted from the measured current and junction temperature data. The results show that both long-term LED illumination and higher intense radiation can result in a larger temperature rise, which needs long-term reverse bias to cool down. Therefore, the applied driving pulse across the LED should be optimized in terms of amplitude and pulse width to avoid temperature rise more than the regulatory limit and cool down the LED’s surface temperature. The thermal decay profile shows three dominant time constants, which we assume they relate to the optrode bulk, LED and its encapsulation.

## Discussion

This work presents a current-mode source measure interface which is practical as a compact LED-based temperature sensor in optogenetics. Such integrated CMOS sensors require small area and immunity to different process variations [37–39].

Prior to this work, some resistor and RTD-based temperature sensors are reported to monitor the temperature in brain, retina and myocardium [11–14]. Goncalves et al. reported an optrode with an integrated temperature sensor based on RTD [14]. The implemented 3D probe has positioned RTD on the opposite side of the LED to reduce the distance between the heating source (LED) and the thermal sensor (RTD). Although RTD sensors have a linear response over a wide range of temperature variation, implementing a resistor requires a considerable area (520 µm × 300 µm). Consequently, employing the LED on the optrode as thermal sensing device can reduce the probe size and accurately measure the LED surface temperature. We used the thermal and electrical properties of LEDs to enable the employment of LED as its own temperature sensor.

In this method, a robust voltage supply is needed to bias the LED, which can be realized with integrated voltage references [40–42], whereas we have used external DACs in our experiments. The critical condition can occur when the LED bias voltage is changing during the sensor operation. In other words, the main challenge is to maintain the bias voltage dependent from the variation in the temperature-sensitive parameter of the sensor. We achieved this via a CCII interface with the required functionalities at a low-power operation compared to the reported designs. We then demonstrated the efficacy of the sensor interface.

The bandwidth of the CCII is enough for low-frequency operations up to 130 kHz where for the temperature sensor application, the pulsing LED does not exceed a few kHz. This limit is introduced by the bias currents which are deliberately kept low to minimize the power consumption. The total measured power consumption of the sensor interface is 260 µW. A summary of specifications comparing the designed CCII with previously reported circuits is listed in Table 2. The design has a high accuracy for the unity gains compared to a similar design in same technology. It also takes benefit of single power supply and low power compared to the designs in advanced technologies.

**Table 2:** CCII specifications compared to the reported designs

Specifications	[30]	[43]	[44]	This work
CMOS tech. (µm)	0.35	0.18	0.25	0.35
Power supply (V)	Dual ± 1.25	Dual ± 0.75	Dual ± 1.5	Single + 5
DC voltage gain	0.96	1.0001	–	0.9826
DC current gain	0.976	1	–	1.0033
Voltage transfer BW (Hz)	3.9G	1.22G	85 M	45 k
Current transfer BW (Hz)	2.6G	1.24G	120 M	130 k
Power (W)	–	268µ	1.74 m	10µ
PSRR (dB)	–	–	41.27	57@10 kHz Δ*V*_dd_ = 1.4 V
THD (%)	–	–	0.9@1 MHz	0.1@10 kHz

Table 3 compares the designed temperature sensors with resistor-based temperature sensors for in vivo application, whereas only one is integrated within the optrode with LEDs for optogenetics application [14]. Our achieved temperature range is based on the measured reverse current change versus temperature variation, which is dependent on the applied bias and mainly the LED type. The designed circuit can use a different type of LED for a wider range of temperature sensing based on their reverse current range and temperature sensitivity. The minimum measured temperature sensitivity is 5 mV/ °C, whereas a 12-bit ADC in processing stage determines a resolution of 0.2 (°C).

**Table 3:** Specification of temperature sensors for in vivo application

Specifications	[11]	[12]	[13]	[14]	This work
Temperature-sensing device	Poly-Si RTD	Au resistor On neural probe	Poly-Si RTD	Pt-based RTD On optrode	LED as a self-sensor
Temperature-sensing parameter	Resistance	Resistance	Resistance	Resistance	LED reverse current
Region of sensing	Brain tissue	Brain tissue and retina	Myocardium	Brain tissue	Brain tissue
Temperature range (°C)	30 to 45	18 to 42	15 to 70	0 to 60	0 to +45
Resolution (°C)	0.1	–	–	0.19	0.2
Complexity and process	Micromachining	Micromachining	Micromachining	Micromachining	CMOS only

The final consideration is that there needs to be a calibration between the junction temperature of the LED and the surface temperature of the probe. We have previously shown this in [9, 15], but as we are not a manufacturing unit, we do not know to what extent the characteristics would vary with scale, i.e. would each probe need to be calibrated separately? Or could a common statistic be used—assuming probe fabrication and encapsulation variability is kept to a minimum. That would need to be determined by a future probe manufacturer.

## Conclusion

In this paper, a CMOS current-mode sensor interface has been presented to measure the temperature variation at the surface of LEDs in optoelectronics. The LED has been employed as a temperature-sensitive element, and its reverse current has been used as TSP to calculate the surface temperature change. This approach effectively reduces the implantable device’s area and its fabrication complexity by reusing the LED on optrode for sensing. Using a low-power CCII to interface the LED has overcome the challenge of converting the LED reverse current change to temperature variation independent of bias voltage variation. The CCII is robust against power supply ripple and transistor mismatch, which makes the circuit reliable as a sensor interface. The achieved results from the circuit characterization and in vivo experiments show the feasibility of the sensor in monitoring the tissue surface temperature in optogenetics.

## Methods

Figure 1b shows the optrode concept: electronics circuits provide control and driving and mini-LEDs provide the optical emission as per [4]. In order to monitor temperature changes, we previously devised a method of monitoring temperature change of the optrode surface by monitoring the current on at the LED junction [45]. The operation can, therefore, be oscillated between stimulating forward driving of the LEDs as shown in Fig. 1c and reverse driving to monitor the temperature-dependent LED current, as shown in Fig. 1d. The forward and reverse phase scheme can be seen in Fig. 1e. The key objective of this work has to develop a circuit to drive and monitor this operation.

During the forward driving phase (Fig. 1c), the LED is turned on (i.e. emits light) and the temperature-sensing circuit is off. Driving the LED will increase heat, which can then be measured in the reverse sensing phase shown in Fig. 1d. During this phase, the LED is off, but the LED needs to be biased at a precise reverse voltage in order to monitor temperature rises compared to ambient. Several LEDs have been investigated theoretically and experimentally to show the linear relationship between the reverse current and surface temperature (*T*_S_) [15, 46, 47]. Figure 1e shows the timing diagram used for switching the LED between the stimulation and sensing phases [48].

**Fig. 7:**
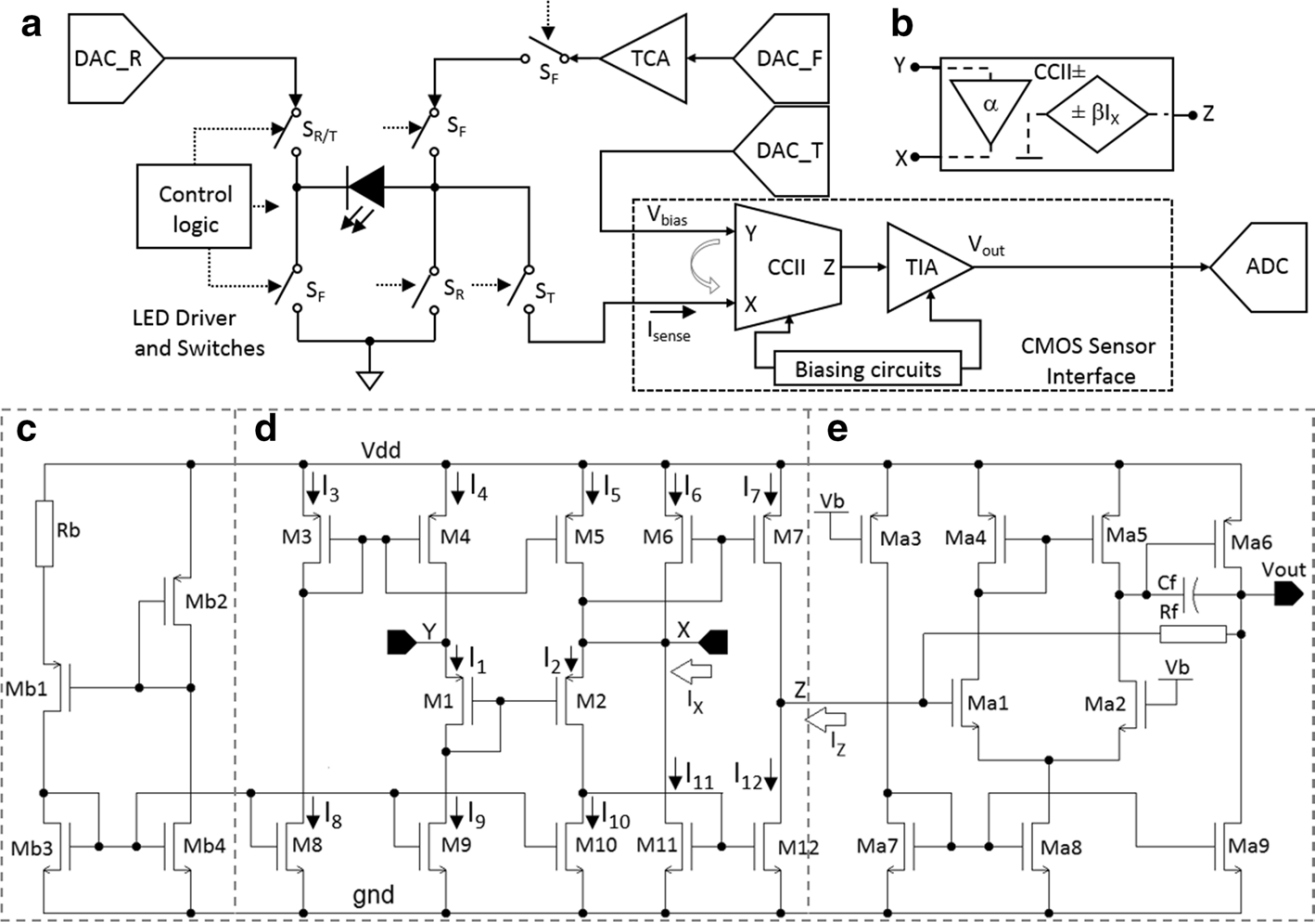
Block diagram of (**a**) the developed sensor interface connected to LED in H-bridge structure. The *S*_F_, *S*_R_ and *S*_T_ switches are controlled to have the LED in forward bias (ON), reverse bias (OFF) and temperature sensing, respectively. **b** Equivalent circuit of CCII consisting of a voltage buffer and a current-controlled current source; the circuit diagram of the sensor interface including, **c** biasing circuit; **d** CCII; and **e** TIA

Figure 7a shows a block diagram of the implemented sensor interface on CMOS which is connected to LED in H-bridge structure. The H-bridge allows the LED to be switched between forward (LED-ON), reverse (LED-OFF) and sensing phases by controlling the S_F_, S_R_ and S_T_ switches, respectively. In order to monitor the increased temperature due to optical stimulation, the LED is switched to the sensor interface path after the forward phase. DAC_F and a transconductance amplifier (TCA) provide the required bias in forward phase. DAC_R is to reversely bias and turn off the LED. In sensing phase, DAC_T controls the LED voltage through a CCII [49] which is buffering the output voltage of DAC_R from Y to X. This voltage remains independent of the received reverse current (*I*_sense_) at X. The current is then transferred to the transimpedance amplifier (TIA) to be converted and amplified for processing.

Figure 7 (b) shows the symbolic representation of the CCII which operates as a voltage buffer from Y to X and current buffer between X and Z terminals. Hence, these characteristics are used to provide and maintain a robust bias voltage for the sensing element connected to X and convey the received current from X to Z terminal. The terminal characteristics of the CCII are presented by the hybrid matrix Equation in (1):

1 $$\left[ {\begin{array}{*{20}c} {V_{{\text{X}}} } \\ {I_{{\text{Y}}} } \\ {I_{{\text{Z}}} } \\ \end{array} } \right] = \left[ {\begin{array}{*{20}c} \alpha & 0 & 0 \\ 0 & 0 & 0 \\ 0 & \pm & 0 \\ \end{array} } \right]\left[ {\begin{array}{*{20}c} {\begin{array}{*{20}c} {V_{{\text{Y}}} } \\ {I_{{\text{X}}} } \\ \end{array} } \\ {V_{{\text{Z}}} } \\ \end{array} } \right]$$

The parameters, *α* and *β*, are frequency-dependent current and voltage gains which are ideally equal to unity. The symbol ± for *β* indicates whether the current conveyor is an inverting or non-inverting circuit, named CCII− or CCII+. A positive sign refers to the same direction for *I*_X_ and *I*_Z_ currents into or from the conveyor.

The CMOS realization of the sensor interface is depicted in Fig. 7c–e, and the aspect ratios of the transistors are listed in Table 4.

**Table 4:** Transistors aspect ratio for CCII and TIA

Design	CCII			TIA					
	PMOS		NMOS	PMOS			NMOS		
MOS	M1-2	M3-7	M8-12	Ma1-2	M7a	M8a–9a	M3a	M4a–5a	M6a
W/L^a^	1/5	1/10	1/25	5/1	15/1	20/1	3/1	15/1	28/1

^a^W and L all are in µm

**Fig. 8:**
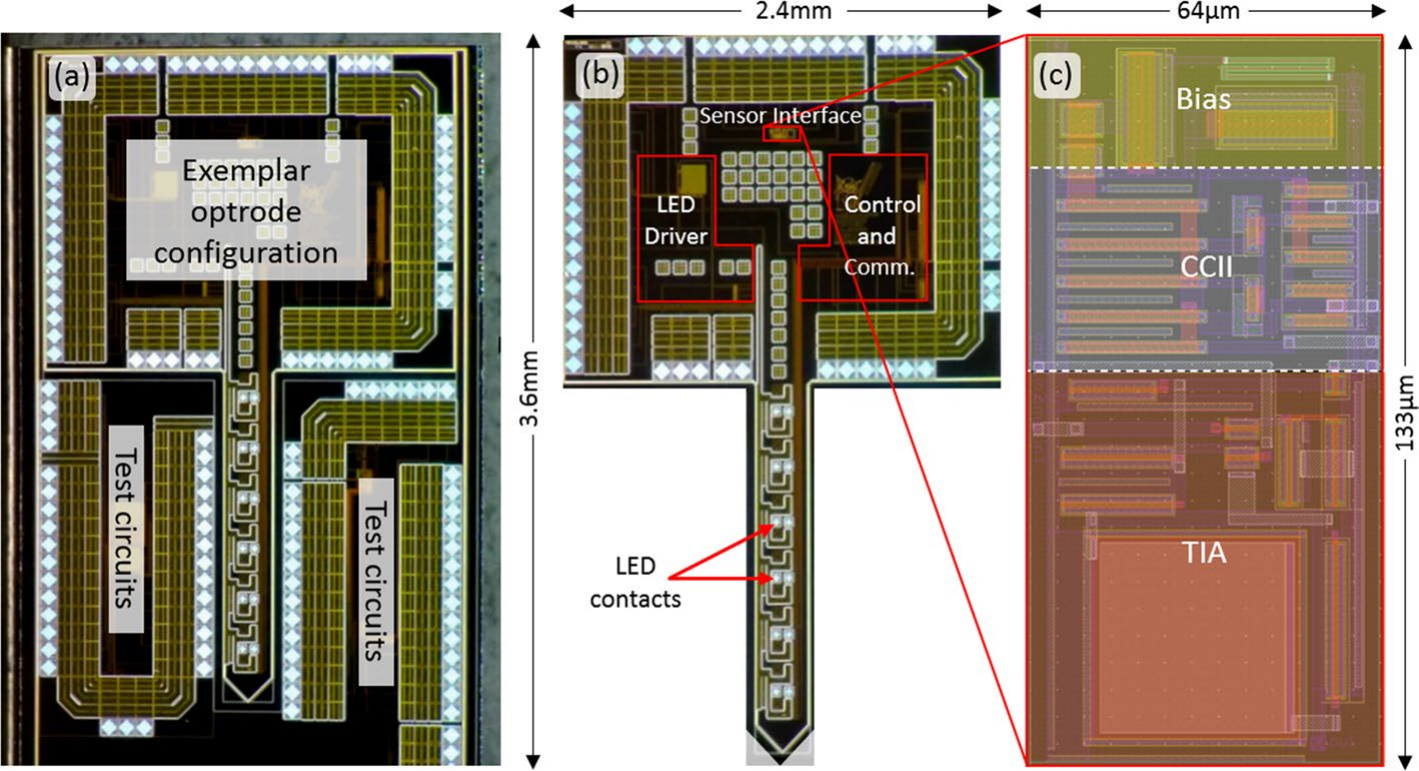
**a** Micrograph of a fabricated chip including an optrode; **b** exemplar developed optrode including LED driver (for optical stimulation), control and communication and sensor interface circuits with LED contacts; and **c** layout of the sensor interface which occupies an area of 64 µm × 133 µm

The biasing circuit provides the bias current and voltage for the CCII and amplifier. The input current is received at X and amplified to an output voltage (*V*_out_). The employed three cascode transistors in the CCII structure allows to scale down the supply while having enough overdrive voltage and dynamic range needed for the design. The input transistors (M1–M2) are matched to have the voltage follower concept from Y to X terminals (*V*_Y_ = *V*_X_). All other PMOS (M3–M7) and NMOS transistors (M8–M12) are matched to eliminate distortion. The relationship between *I*_Z_ and *I*_X_ can be expressed as:

2 $$I_{1} \sim I_{5} = I_{8} \sim I_{10} ,\;I_{6} = I_{7} , I_{11} = I_{12} \Rightarrow I_{X} = I_{Z}$$

where *I*_n_ is the bias current of the transistor *M*_n_. The circuit is capable of detecting and conveying small currents in the range of hundreds of pA to 10 of µA. This range covers the output current of different current-mode sensors. The input and output common-mode range of the circuit varies around Vdd/2, and the bias voltage of Vdd/2 ± Δ*V* at Y is followed at X while *I*_X_ is being conveyed to Z terminal. The circuit is using 5 V supply voltage to provide the required common-mode voltage at X terminal for the LED, which was targeted as the main current-mode sensor. Optical stimulation needs enough light intensity to stimulate the nerve cells, which means enough power for the LED and therefore enough bias voltage on the LED for illumination. The employed LED is biased using 5 V supply voltage in the stimulation phase to generate enough light illumination for optical stimulation. Therefore, designing the CCII with the same supply voltage enables a proper range of common-mode voltage at X and the possibility to apply the needed *V*_bias_ for the target reverse bias voltage on the LED. The designed amplifier is a traditional differential structure with a transimpedance amplification gain of 10^6^ (V/A). Figure 8a shows a micrograph of the fabricated chip which includes an exemplar optrode. Figure 8b shows the CMOS electronics and LEDs on the optrode. The layout of the sensing circuits is depicted in Fig. 8c.

## Data Availability

The datasets related to the current study are available from the corresponding author on reasonable request.

## Abbreviations

LED: light emitting diode
CCII: second-generation current conveyor
RTD: resistance temperature detector
TSP: temperature-sensitive parameter
CMRR: common-mode rejection ratio
THD: total harmonic distortion
PSRR: power supply rejection ratio
MC: Monte Carlo
NHP: non-human primate
SMU: source measure unit
TCA: transconductance amplifier
TIA: transimpedance amplifier
